# Plasma Metabolic Profile with Machine Learning Reveals Distinct Diagnostic and Biological Signatures for Pathologic Myopia

**DOI:** 10.1002/advs.202505861

**Published:** 2025-07-25

**Authors:** Ziheng Qi, Jiao Qi, Ye Zhang, Yanhui Wang, Yuchen Feng, Zifan Yang, Yating Wang, Weikang Shu, Dongling Guo, Ching Kang, Keke Zhang, Yi Lu, Jingjing Wan, Xiangjia Zhu

**Affiliations:** ^1^ School of Chemistry and Molecular Engineering East China Normal University Shanghai 200241 P. R. China; ^2^ Eye Institute and Department of Ophthalmology Eye & ENT Hospital, Fudan University Shanghai 200031 P. R. China; ^3^ NHC Key Laboratory of Myopia and Related Eye Diseases Key Laboratory of Myopia and Related Eye Diseases Chinese Academy of Medical Sciences Shanghai 200031 P. R. China; ^4^ Shanghai Key Laboratory of Visual Impairment and Restoration Shanghai 200031 P. R. China; ^5^ State Key Laboratory of Medical Neurobiology Fudan University Shanghai 200031 P. R. China

**Keywords:** machine learning, mass spectrometry, metabolic biomarker, myopic macular degeneration, pathologic myopia

## Abstract

Pathologic myopia (PM), characterized by serious myopic macular degeneration (MMD), is a detrimental subtype of high myopia (HM) and has become one of the leading causes of blindness worldwide. In this concern, precise and high‐throughput molecular diagnosis and further pathologic insights are urgently needed. Here, through the combined strategy of nanoparticle‐enhanced laser desorption/ionization mass spectrometry‐based rapid metabolic analysis (<30 s) and machine learning, a precise molecular diagnostic approach of PM (HM with MMD grade ≥ 2) is proposed, which achieves areas under the curve of 0.874 and 0.889 for diagnosing PM and early‐stage PM, respectively. Further, the biomarkers indicate the PM‐associated systemic metabolic reprogramming of amino acid and lipid metabolism, which may mediate dysfunctional oxidative stress, inflammation, hormone/neurotransmitter systems, and energy metabolism. Notably, MMD grade 4, featuring characteristic macula atrophy, exhibits specificity in this metabolic reprogramming. Of these biomarkers, azelaic acid shows a significant protective effect in the ARPE‐19 cells under abnormal oxidative stress, which may be involved in PM development as a key antioxidative active metabolite. This work will contribute to PM molecular diagnosis and pathology exploration.

## Graphical abstract

Plasma metabolic detection combined with machine learning offers a precise (area under the curve of 0.874) and high‐throughput (<30 s) diagnosis for pathologic myopia (serious myopic macular degeneration). The biomarkers indicate the pathologic myopia‐associated systemic metabolic reprogramming, which exhibits specificity for myopic macular degeneration grade 4. Of these, azelaic acid may be involved in pathologic myopia development as a key antioxidative active metabolite.

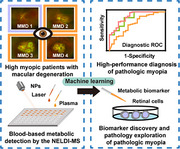

## 1 Introduction

With a remarkable surging prevalence, high myopia (HM) has emerged as a significant public health concern that impacts ≈400 million individuals.^[^ ^1^ ^]^ Thereinto, pathologic myopia (PM), characterized by the presence of typical fundus lesions, is the most detrimental subtype of HM.^[^ ^2^ ^]^ It has been reported that PM is one of the leading causes of blindness worldwide, especially in Asian countries, and poses a considerable economic burden globally, which urgently requires particular concern.^[^ ^3^ ^]^

The definition of PM primarily depends on the degree of myopic macular degeneration (MMD).^[^ ^4^ ^]^ According to the internationally recognized Meta‐Analysis for Pathologic Myopia (META‐PM) Classification System, MMD is classified from grades 0 to 4 based on the severity of retinal and choroidal atrophy lesions.^[^ ^5^ ^]^ HM eyes with MMD 0–1 are defined as simple HM, which means their fundus is relatively healthy. Meanwhile, eyes with MMD ≥ 2 are defined as PM, which may significantly impair the vision. Among them, MMD 4, which mainly causes macular atrophy, can lead to even significant visual impairment or blindness. Accurate diagnosis of PM is an essential prerequisite for guiding the visual prognosis of HM patients. Currently, clinical grading of MMD mainly relies on clear fundus photographs. However, while handheld fundus photography equipment is not expensive, it is still not widely available in primary general practice facilities, which support the daily medical needs of large segments of the population in developing countries. And, the handheld fundus photography equipment‐based examination typically requires several minutes of an ophthalmologist's operation time, rendering such equipment ill‐suited for large‐scale screening initiatives in primary care settings. On the other hand, under the other conditions where fundus images are not available, like eye diseases such as corneal or lens opacity prevent the acquisition of clear fundus images, the diagnosis of PM will become very difficult. Moreover, fundus photograph‐based diagnosis highly depends on the experience of professional doctors and is highly subjective. In particular, accurately identifying early‐stage PM (MMD 2) is rather challenging even for professionals. Therefore, there is still an unmet clinical need for a highly accessible, objective, non‐expertise‐dependent PM molecular diagnosis approach with the applicability for widespread screening.

Metabolites as the end of biological pathways are directly related to phenotype changes, unlike nucleic acids and proteins.^[^ ^6^ ^]^ The identification of disease‐associated metabolic reprogramming promises effective molecular diagnostics and novel insights into the development mechanism of diseases.^[^ ^6b^ ^]^ Recently, previous studies have indicated that myopia is not merely an ocular condition but also a systemic disorder with dysregulated metabolic programming as well.^[^ ^7^ ^]^ Blood metabolic reprogramming is likely critically involved with PM development but has not yet been elucidated, which is of great value in aiding mechanism understanding and further research on treatment and prevention approaches. More importantly, considering blood can be easily collected in any primary care facility, blood test‐based metabolic molecular diagnosis of PM can solve the current constraints of PM diagnosis via the network of central laboratories and primary medical institutions and become a potential high‐accessibility approach for large‐scale PM screening in HM patients.

Mass spectrometry (MS) represents the primary tool for metabolic analysis, with strong capabilities of broad molecular profiling and identification through measuring the mass‐to‐charge ratio (m/z).^[^ ^8^ ^]^ However, in the conventional MS‐based metabolic analysis, chromatographic separation remains a necessary step for reducing matrix effects and ionization suppression of complex biological samples, which limits the analysis throughput due to its typical requirement of tens of minutes for pretreatment and separation.^[^ ^9^ ^]^ In contrast, nanoparticle‐enhanced laser desorption/ionization MS (NELDI‐MS) allows high‐performance solid‐phase metabolic profiling without the reliance on chromatographic separation or other complex pretreatments, as an emerging high‐throughput tool for metabolic analysis aiming to aid diagnosis.^[^ ^10^ ^]^ To date, the NELDI‐MS‐based strategy that integrates metabolic fingerprints and machine learning has discovered the metabolic reprogramming of a wide range of diseases,^[^ ^10^, ^11^ ^]^ encouraging us to try NELDI‐MS to reveal PM‐associated blood metabolic reprogramming and construct a rapid molecular diagnostic approach suitable for large‐scale screening.

**Scheme 1:**
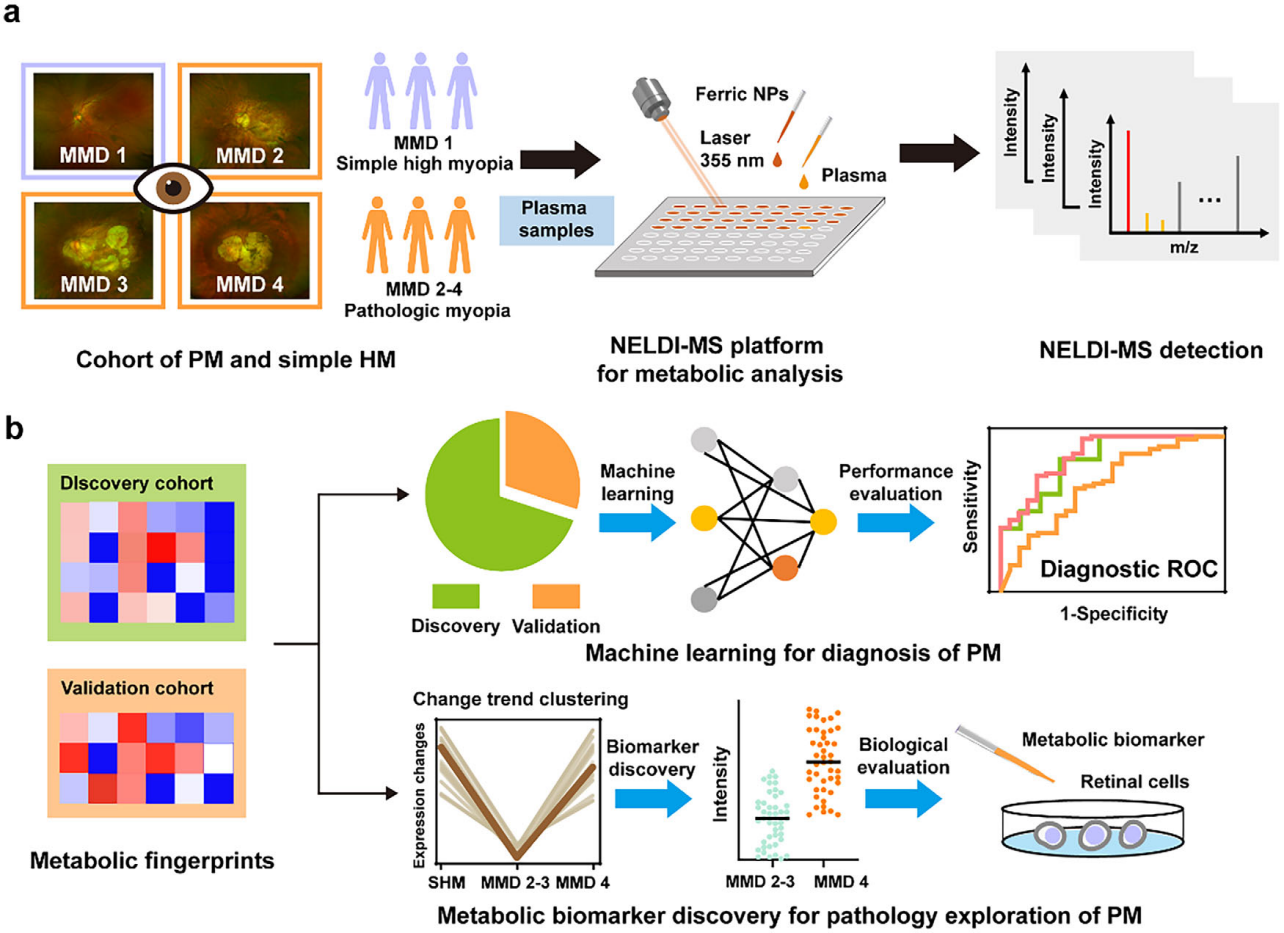
Schematic for characterizing the plasma metabolic reprogramming of PM for rapid diagnostic and pathology exploration. a) We collected plasma samples from the HM subjects with MMD (35 simple HM subjects with MMD 1 and 90 PM subjects with MMD 2–4, typical fundus pathology was shown). Using the high‐performance NELDI‐MS platform, we performed metabolic analysis for the plasma samples by automatic rapid MS detection. b) We constructed the PMFs of those plasma samples from the original MS data, supporting the applications of diagnosis and pathology exploration through efficient data parsing for the PMFs.

Herein, we performed rapid metabolic analysis (<30 s per sample) by the NELDI‐MS for the plasma samples from 125 HM subjects with MMD (35 simple HM subjects with MMD 1 and 90 PM subjects with MMD 2–4), and thus constructed a plasma metabolic fingerprint (PMF) ‐based machine‐learning diagnostic model of PM (**Scheme** **1**). The model achieved an area under the curve (AUC) of 0.874 for diagnosing PM, without significant performance decline for diagnosing early‐stage PM (AUC of 0.889), i.e., HM eyes with MMD 2. On this basis, we further identified biomarkers from the PMFs, which indicated the distinctive systemic metabolic reprogramming regarding PM and highlighted the specificity of MMD 4. Of these biomarkers, we found azelaic acid (AZA) may be involved in PM development as a key antioxidative active metabolite. Our findings offer a desirable option for large‐scale PM screening, and provide insights into its pathogenesis.

## 2 Result

### 2.1 NELDI‐MS enables High‐Performance Metabolic Analysis

**Figure 1:**
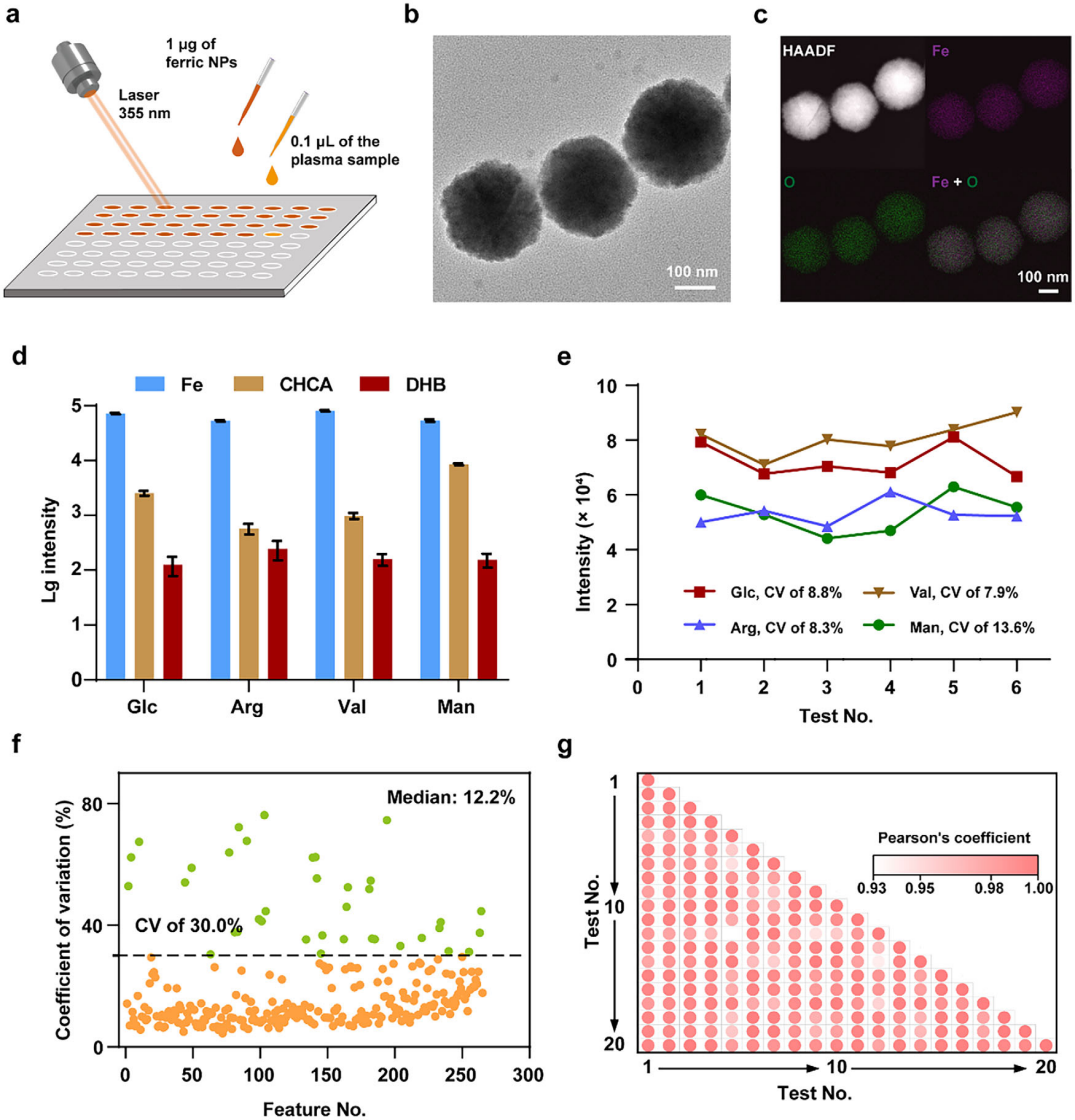
NELDI‐MS enables high‐performance metabolic analysis. a) The illustration of the NELDI‐MS. b) Transmission electron microscopy image of the ferric NPs (scale bar of 100 nm). c) The high‐angle annular dark field (HAADF) and elemental mapping analysis for the ferric NPs (scale bar of 100 nm). d) Comparison of the LDI‐MS intensity of the four metabolite standards among the use of NPs, 2,5‐dihydroxybenzoic acid (DHB), and *α*‐cyano‐4‐hydroxy‐cinnamic acid (CHCA). The standards included glucose (Glc), arginine (Arg), valine (Val), and mannitol (Man). The data represented six independent repeated tests and was expressed as the mean ± standard error. e) CVs of the MS intensity of the four metabolite standards in the six independent repeated NELDI‐MS tests. f) CVs of the 265 m/z features detected from the representative plasma sample by the NELDI‐MS in the 20 independent repeated detections. The median CV was 12.2%. The dotted line represented the CV of 30.0%, and 228 features had a CV less than that. g) Pearson correlation coefficients among the 20 independent repeated detections for the representative plasma sample.

To record the PMFs of HM subjects with MMD, we constructed a high‐performance NELDI‐MS platform based on the ferric oxide nanoparticles (NPs) for metabolic analysis (**Figure** **1**a). In the preparation of the NPs, an optimized solvothermal workflow was employed, according to our previous works.^[^ ^11a^ ^]^ For characterization, the Fe_2_O_3_ crystal structure of the NPs was confirmed by X‐ray diffraction (Figure S1, Supporting Information). Further, we characterized the morphology of the NPs using transmission and scanning electron microscopy (Figure 1b; Figure S2, Supporting Information), and determined the uniform distribution of the Fe and O elements on the NPs using elemental mapping analysis (Figure 1c). In a typical process of metabolic analysis, 1 µg of the NPs was loaded onto each sample spot formed by 0.1 µL of plasma (Figure 1a), allowing the automatic detection for up to 384 microarrayed sample spots with high throughput, in which the detection can be finished within 30 s per sample.

In terms of analysis performance, we employed four typical standard small metabolites to evaluate the detection sensitivity and reproducibility of the NELDI‐MS platform. For sensitivity, compared to the commonly used commercial organic matrices, the NELDI‐MS exhibited 1 to 3 orders of magnitude enhancements of the MS signal response in the detection for those standard metabolites (Figure 1d), supporting the in‐depth metabolic analysis in clinical plasma samples. We also observed this sensitive detection for several metabolic molecular structures in a mixed solution of high salt and protein (15 mm of Na^+^, 0.5 mm K^+^, and 10 mg mL^−1^ of protein of bovine serum albumin), which suggested the capability of the NELDI‐MS to efficiently metabolic profile from biological matrices without complex pretreatment (Figure S3, Supporting Information). Of note, the NELDI‐MS achieved desirable reproducibility in the six independent detections with coefficients of variation (CVs) of 7.9% to 13.6% (Figure 1e), which is significant for credible metabolic information extraction.

The metabolic analysis performance of the NELDI‐MS platform was further validated by the representative plasma sample, which was prepared by mixing five samples from each MMD grade. Of note, for pretreatment, the raw plasm sample only needed a simple 10‐fold dilution in water as the reported procedure,^[^ ^12^ ^]^ due to the tolerance of the NELDI‐MS for high salt and protein. The pretreatment and detection for this representative sample were independently repeated 20 times, where the NELDI‐MS efficiently recorded ≈120 000 MS original data points at 100–1000 Da in each detection. Using the peak detection algorithm based on the localized highest intensity, we extracted the in‐depth PMFs (more than 250 m/z features, Figure 1f) from the original MS data. Importantly, in the 20 repeated MS detections, the median of CVs of all m/z features is 12.2%, and the CVs of more than 85% of m/z features were below 30%, as well as the 20 PMFs showed a range of 0.93 to 1 in Pearson correlation coefficients (Figure 1g), demonstrating the reproducibility of metabolic detection. In short, the above results demonstrated the capability of our NELDI‐MS platform for high‐throughput recording PMFs of HM subjects with MMD, with high performance in detection depth and reproducibility.

### 2.2 Design and PMF Acquisition for the PM and Simple HM Cohort

**Figure 2:**
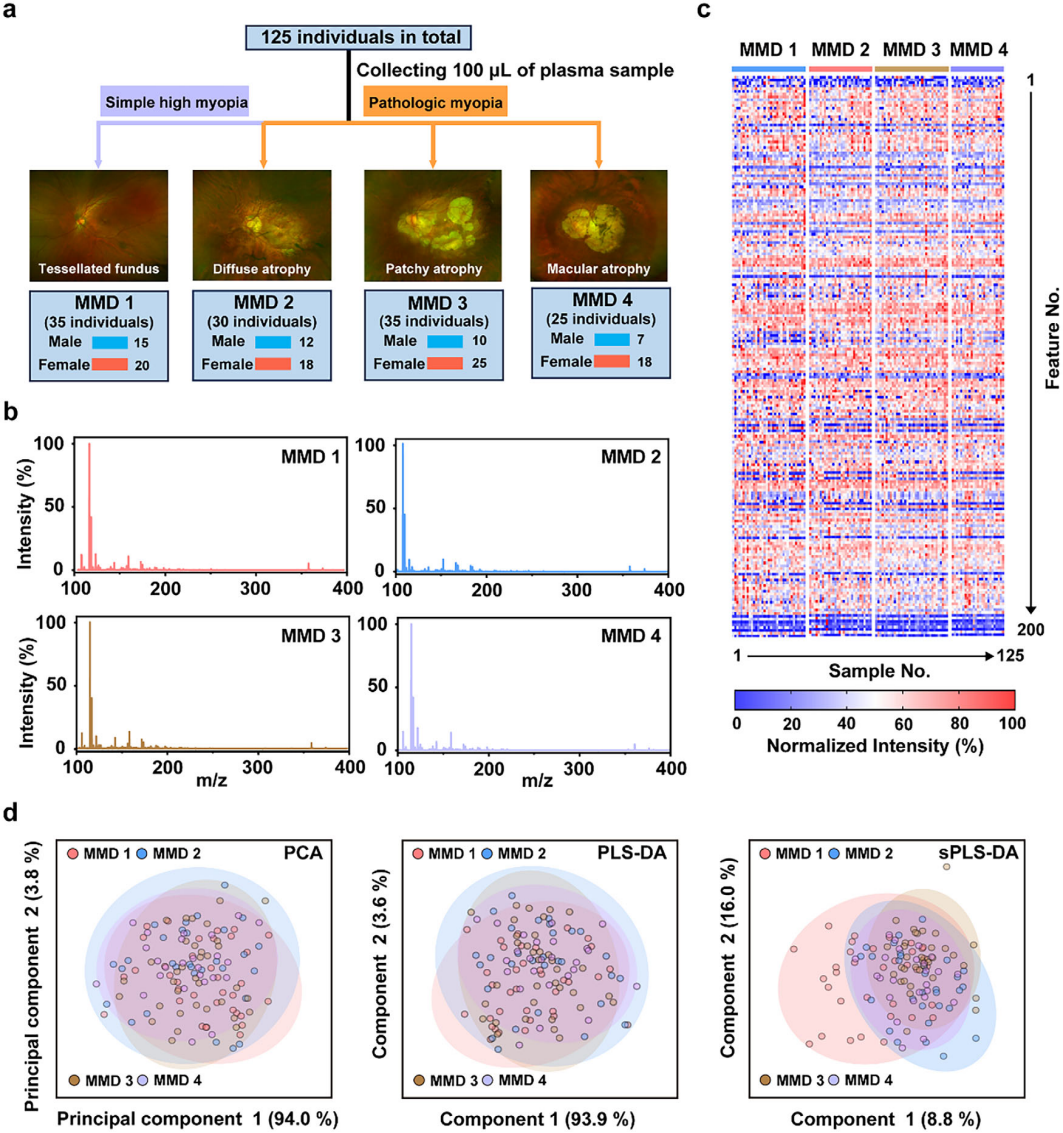
Design and PMF acquisition for the PM and simple HM cohort. a) The design, typical fundus pathology, and basic information of the cohort, including 35 subjects with simple HM (all MMD 1) and 90 subjects with PM (30 subjects with MMD 2, 35 subjects with MMD 3, and 25 subjects with MMD 4). b) Typical MS spectra obtained from the plasma samples of MMD 1 to 4. c) Blueprint of the 125 PMFs, each of which contained 200 m/z features. d) Dimensionality reduction chemometric analysis for discriminating the PMFs of different grade MMD. PCA, principal component analysis, PLS‐DA, partial least squares‐discriminant analysis, sPLS‐DA, sparse PLS‐DA.

To investigate the specific blood metabolic reprogramming of PM, we collected plasm samples from 125 HM subjects with MMD 1–4 (main cohort, **Figure** **2**a), supporting PMF database construction. Specifically, 35 subjects with simple HM (all MMD 1) and 90 subjects with PM (30 subjects with MMD 2, 35 subjects with MMD 3, and 25 subjects with MMD 4) were included in this cohort. The diagnosis and grading of the MMD were made according to the fundus photographs. The demographic characteristics of the included subjects were summarized in Table S1 (Supporting Information), including age, sex assigned at birth, and average axial length of both eyes.

Based on the NELDI‐MS platform, we performed high‐performance metabolic analysis for the plasm samples to construct a PMF database of PM and simple HM. Specifically, the MS data acquisition for the 125 samples was finished within 2 h, where strong MS signals at 100–1000 Da of each sample were recorded and mostly concentrated at the low mass range of 100–400 Da (Figure 2b). After peak detection from the original MS data, we obtained 125 PMFs containing 200 m/z features and built a blueprint to show their distribution of relative intensities (Figure 2c). We next attempted to employ common dimensionality reduction chemometric approaches, including principal component analysis, partial least squares‐discriminant analysis, and sparse partial least squares‐discriminant analysis, to discriminate the PMFs of different grade MMD, which did not show clear separation (Figure 2d). Besides, the unsupervised clustering for the PMFs also failed to separate those of different grade MMD (Figure S4, Supporting Information), suggesting the need for further machine learning modeling.

### 2.3 Machine Learning based on PMFs Achieved Precise Molecular Diagnosis of PM

**Figure 3:**
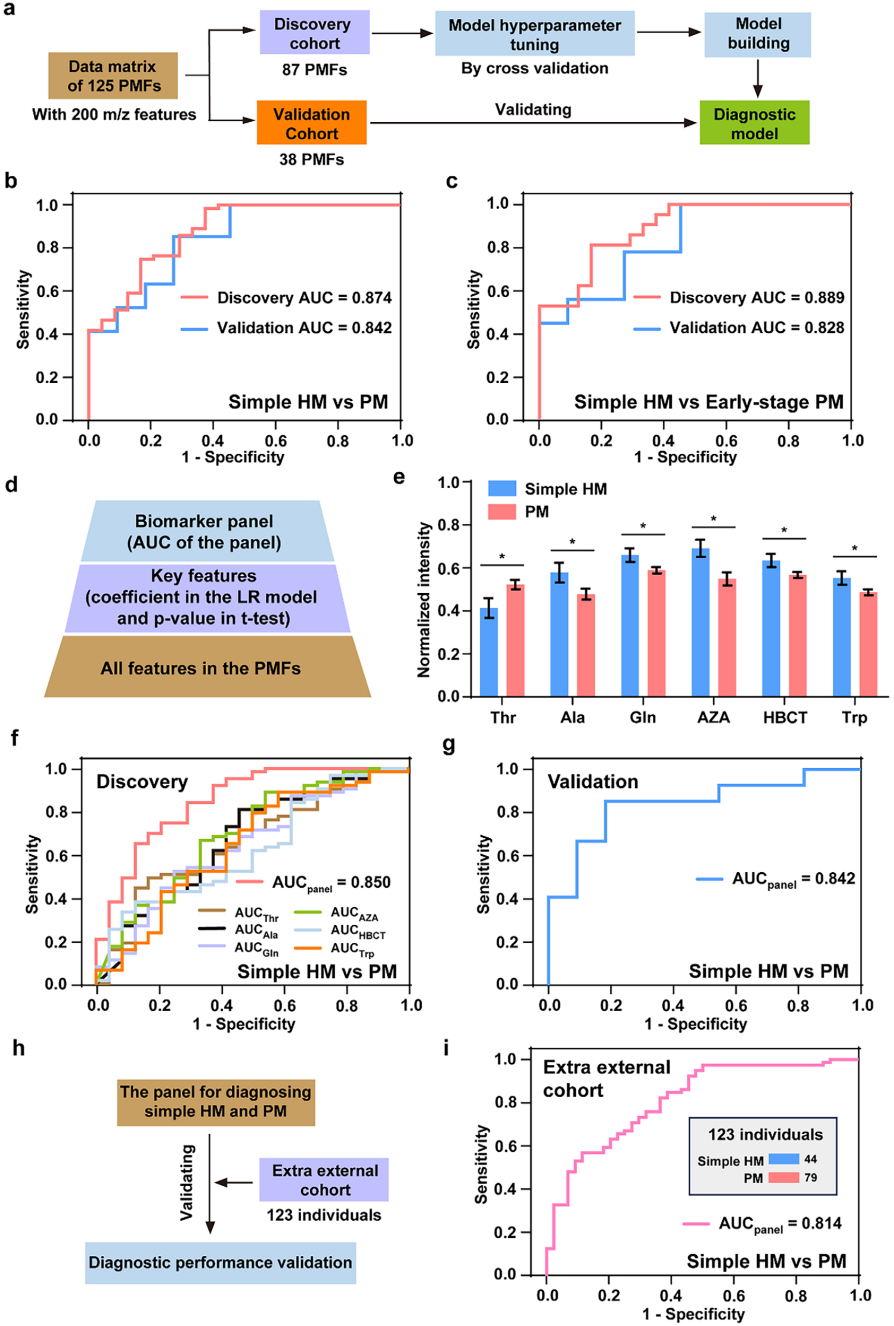
Machine learning based on PMFs achieved precise molecular diagnosis of PM. a) The workflow for building the machine learning model for diagnosing PM based on the PMFs. b) The receiver operating characteristic (ROC) curves by the PMF‐based diagnostic LR model for discriminating PM and simple HM in the discovery cohort (simple HM/PM, 24/63) and validation cohort (simple HM/PM, 11/27). c) The ROC curves by the PMF‐based diagnostic LR model for discriminating early‐stage PM (MMD 2) and simple HM in the discovery cohort (simple HM/early‐stage PM, 24/21) and validation cohort (simple HM/early‐stage PM, 11/9). d) The workflow for constructing the diagnostic biomarker panel from the PMFs. e) The differences between the simple HM and PM groups of the six features making up the panel, which were further annotated as metabolites including Thr, Ala, Gln, AZA, HBCT, and Trp. The bar graph represented the data in the discovery cohort (87 PMFs, from 24 simple HM subjects and 63 PM subjects). The data was expressed as the mean ± standard error and * represented p‐values <0.05 in the two‐tailed t‐test. f) When using the feature panel or single features, the different ROC curves by the diagnostic LR model for discriminating PM and simple HM in the discovery cohort. g) When using the feature panel, the ROC curve by the diagnostic LR model for discriminating PM and simple HM in the validation cohort. h,i) The extra external cohort‐based feature panel validation workflow (in h) and corresponding ROC curve (in i) for discriminating PM and simple HM, which included 44 simple HM subjects and 79 PM subjects.

To achieve the distinction between PM and simple HM, we performed machine learning modeling based on the PMFs to identify its distinctive systemic metabolic reprogramming. Before machine learning, we performed power analysis of the pilot data (PMFs from five simple HM subjects and five PM subjects), which reached a predicted power of more than 0.8 at a sample size of 30 per group (FDR = 0.15, Figure S5, Supporting Information). In terms of machine learning algorithms, we used classic logistic regression (LR) with L1‐ or L2‐ regularization for building the diagnostic model, due to its interpretability rewarding for clinical applications. Before model building, we applied a random split for the 125 PMFs in the main cohort into the discovery cohort (70%) and validation cohort (30%, **Figure** **3**a). In the following machine learning, the discovery cohort was employed to carry out cross‐validation for the model hyperparameter tuning and final model building, while the validation cohort was employed only in model performance testing to exclude the overfitting risk (Figure 3a).

In terms of PM diagnosis performance, the LR model with the best‐tuned hyperparameters afforded an encouraging area under the curve (AUC) of 0.874 (95% CI of 0.789 to 0.958, p‐value <0.001), a sensitivity of 85.7%, and a specificity of 70.8% in the discovery cohort (Figure 3b). Importantly, a validation AUC of 0.842 (95% CI of 0.700 to 0.984, p‐value <0.01) suggested no unacceptable overfitting of the model. Further, we evaluated the performance of the LR model when distinguishing early‐stage PM (MMD 2) from simple HM, which was significant for early detection and early intervention of PM in the clinic. Notably, the discovery AUC of 0.889 (95% CI of 0.798 to 0.980, p‐value <0.001, Figure 3c), corresponding to a sensitivity of 85.7% and a specificity of 70.8%, indicated the applicability of the LR model for diagnosing early‐stage PM, which was also validated in the validation cohort with an AUC of 0.828 (95% CI of 0.649 to 1.000, p‐value <0.05).

Next, we tried to construct a diagnostic biomarker panel from the PMFs, considering metabolic complexity may limit potential clinical translation. For feature selection, the features were ranked by their coefficients in the LR model by the PMFs, and those with the TOP 30 coefficients were selected as the key features (Figure 3d). Of note, the features without significant group differences in MS intensities (significance level was set at 0.05, two‐tailed t‐test) were excluded. Then, we evaluated the diagnostic performance of all combinations of the key features (the number of features in combinations increased one by one) and identified a panel consisting of six features (Figure 3e; Tables S2 and S3, Supporting Information). Those features were further annotated as metabolites using ultra‐high‐resolution Fourier transform ion cyclotron resonance (FTICR) ‐MS and LC‐MS/MS, including threonine (Thr), alanine (Ala), glutamine (Gln), tryptophan (Trp), AZA, and hydroxybutyrylcarnitine (HBCT). The Kendall's correlation analysis for these biomarkers showed six significant correlative relationships (p‐values <0.1, Figure S6, Supporting Information). Importantly, the LR model by the panel achieved an enhanced AUC of 0.850 (95% CI of 0.755 to 0.946, p‐value <0.001, corresponding to a sensitivity of 84.1% and a specificity of 70.8%) in the discovery cohort, compared to the limited AUCs of 0.626 to 0.695 when those features were singly employed (Figure 3f). In the validation, the panel also reached a similar AUC of 0.842 (95% CI of 0.709 to 0.975, p‐value <0.01, Figure 3g), excluding the risk of unacceptable overfitting. These results indicated that the six biomarkers making up the panel could represent the metabolic signature and had enhanced applicability for clinical diagnosis.

To further evaluate the biomarker panel's diagnostic performance's reproducibility and clinical translation potential at a larger‐scale level, we included 123 HM subjects with MMD (MMD 1: 44 subjects, MMD 2: 36 subjects, MMD 3: 25 subjects, MMD 4: 18 subjects) as an extra external cohort (Figure 3h; Table S4, Supporting Information). As shown in Figure 3i, the biomarker panel‐based LR model afforded a significant discrimination for simple HM and PM with an AUC of 0.814 (95% CI of 0.736 to 0.893, p‐value <0.001) in this cohort. On this basis, to exclude unacceptable risk of bias, we evaluated the biomarker panel‐based LR model's diagnostic performance in the stratifications of relatively young subjects (Figure S7, Supporting Information) and early‐stage PM subjects (Figure S8, Supporting Information) in both the main cohort and extra external cohort, which afforded AUCs of 0.804 to 0.909 (all p‐values <0.05). These results demonstrated the reproducibility of the biomarker panel's diagnostic performance. Altogether, through rapid high‐performance metabolic analysis (<30 s) by NELDI‐MS for 100 nL of plasma samples and machine‐learning‐based data parsing, we identified the distinctive systemic metabolic reprogramming regarding PM, proposing a promising PM molecular diagnostic approach suitable for large‐scale screening.

### 2.4 Identification of the Specific Systemic Metabolic Reprogramming of MMD 4

As mentioned above, though MMD of grades 2–4 is considered PM, MMD 4 features characteristic fundus pathology of significant macula atrophy, whereas MMD 2–3 are not involved in macula, which encouraged us to investigate their differential systemic changes in metabolites to contribute to molecular mechanism exploration. For this purpose, the 125 PMFs were assigned to three groups in the following study, including the simple HM group (MMD 1), MMD 2–3 group, and MMD 4 group.

**Figure 4:**
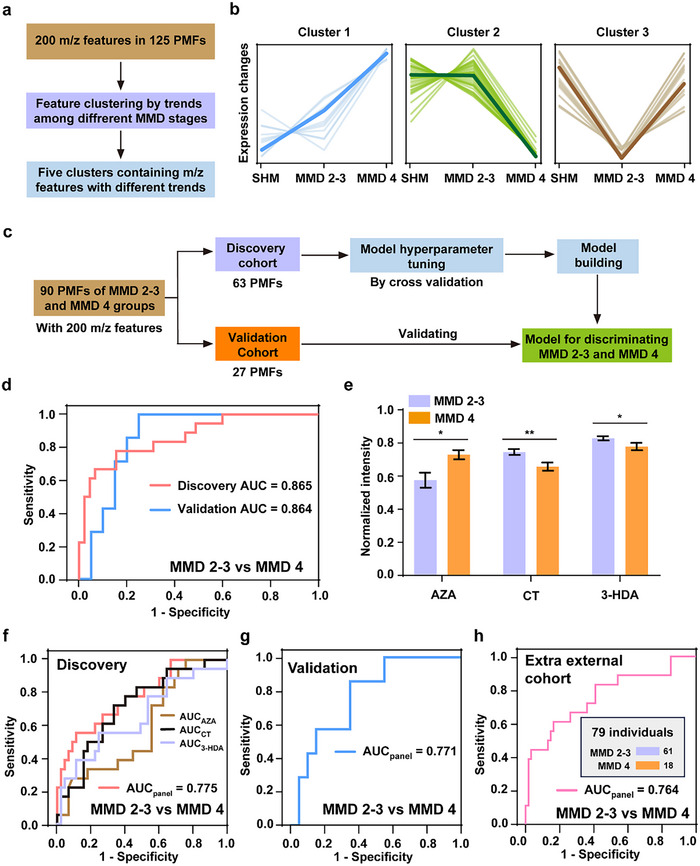
Identification of the specific systemic metabolic reprogramming of MMD 4. a) The workflow for clustering analysis for the 200 features to identify their typical changing trends among the simple HM group, MMD 2–3 group, and MMD 4 group. SHM, simple HM. b) The clusters each represented a typical type of change trend of the features. Three out of the five clusters were shown here, while the other two clusters were shown in the Supporting Information. c) The workflow for building the machine learning model for discriminating MMD 2–3 and MMD 4 based on the PMFs. d) The ROC curves by the PMF‐based classification LR model for discriminating MMD 2–3 and MMD 4 in the discovery cohort (MMD 2–3/MMD 4, 45/18) and validation cohort (MMD 2–3/MMD 4, 20/7). e) The differences between the MMD 2–3 group and MMD 4 group of the three features making up the panel, which were further annotated as metabolites including AZA, CT, and 3‐HDA. The bar graph represented the data in the discovery cohort (63 PMFs, from 45 MMD 2–3 subjects and 18 MMD 4 subjects). The data was expressed as the mean ± standard error. * and ** represented p‐values <0.05 and p‐value <0.01 in the two‐tailed t‐test, respectively. f) When using the feature panel or single features, the different ROC curves by the classification LR model for discriminating MMD 2–3 and MMD 4 in the discovery cohort. g) When using the feature panel, the ROC curve by the classification LR model for discriminating MMD 2–3 and MMD 4 in the validation cohort. h) In the extra external cohort‐based validation for the feature panel, the ROC curve for discriminating MMD 2–3 and MMD 4, which included 61 MMD 2–3 subjects and 18 MMD 4 subjects.

To observe the metabolic changes, we conducted a clustering analysis for the 200 features within the PMFs, based on the changing trends of those features among the three groups (**Figure** **4**a). Those features were included in five clusters (Figure 4b; FigureS9, Supporting Information), each showing a typical type of change trend of the features. Of note, though three of the five clusters showed consistently up‐regulated or down‐regulated trends in the two PM groups (MMD 2–3 group and MMD 4 group, clusters 1, 4, and 5), the trends represented by the other two clusters exhibited interesting differences (clusters 2 and 3). Specifically, cluster 2 indicated some features had strong down‐regulated trends in only the MMD 4 group, compared to the simple HM group. Cluster 3 represented that some features, with similar levels between the simple HM group and MMD 4 group, were decreased trends in only the MMD 2–3 group. These findings suggested the specific systemic metabolic reprogramming of MMD 4, which may be related to its characteristic fundus pathology of significant macula atrophy.

Further, using machine learning with the LR algorithm, we evaluate the predictive power of this systemic metabolic reprogramming. A total of 90 PMFs, from the MMD 2–3 group (negative label) and MMD 4 group (positive label), were included in the machine learning, which contained 63 PMFs in the discovery cohort and 27 PMFs in the validation cohort (Figure 4c). Based on the discovery cohort, we determined the best‐tuned modeling hyperparameters by cross‐validation and built the LR model, which achieved a strong discriminating performance with an AUC of 0.865 (95% CI of 0.766 to 0.965, p‐value <0.001, Figure 4d), a sensitivity of 77.8%, and a specificity of 84.4%. And, the model reached a similar performance in the validation cohort, with an AUC of 0.864 (95% CI of 0.728 to 1.000, p‐value <0.01). The strong predictive power of the PMFs highlighted the specificity of MMD 4‐associated systemic metabolic reprogramming.

On this basis, we further tried to use a small number of metabolites to represent this systemic metabolic reprogramming, thereby constructing a panel consisting of three features based on the previously described workflow (Figures 3d and 4e; Tables S5 and S6, Supporting Information) and annotating those as metabolites by ultra‐high resolution FTICR‐MS and LC‐MS/MS, which included AZA, carnitine (CT), and 3‐hydroxydodecanoic acid (3‐HDA). Of these, CT showed significant Kendall's correlations with 3‐HDA (p‐values <0.1, Figure S10, Supporting Information). The LR model by the panel implemented the significant distinction between the MMD 2–3 group and MMD 4 group (Figure 4f,g; discovery AUC of 0.775, p‐value <0.001, 95% CI of 0.647 to 0.903; validation AUC of 0.771, p‐value <0.05, 95% CI of 0.586 to 0.957), demonstrating those three biomarkers represented the signature of this metabolic reprogramming. Further, this biomarker panel was validated in the extra external cohort (including 79 individuals with MMD 2–3 or MMD 4), which reached a consistent AUC of 0.764 (Figure 4h, p‐value <0.001, 95% CI of 0.628 to 0.900), showing our findings' reproducibility.

For metabolic pathway, as a medium‐chain *β*‐hydroxy fatty acid, 3‐HDA mainly requires conjugation with CT to form ​3‐hydroxydodecanoylcarnitine​ to transport into the mitochondria and undergo *β*‐oxidation.^[^ ^13^ ^]^ AZA, as a dicarboxylic fatty acid, could be generated by *ω‐*oxidation of monocarboxylic fatty acids, and participates in the CT‐dependent pathway to form nonanedioylcarnitine for mitochondrial import or engages in the CT‐independent pathway for other functions like anti‐oxidation.^[^ ^14^ ^]^ In sum, we found the specific systemic metabolic reprogramming of MMD 4 involving CT and fatty acid metabolism, and identified the involved key differential metabolites.

### 2.5 AZA Protects ARPE‐19 Cells from Oxidative Stress

Metabolic biomarkers represent the signature of metabolic reprogramming and exerted various physiological effects in life activities, which might play key roles in PM development. In this regard, of the biomarkers identified here, AZA aroused our great interest in further investigating its bioactivity, due to its presence in both the two biomarker panels, reported activity in mitigating oxidative stress,^[^ ^14a^ ^]^ and reported relation to myopia.^[^ ^15^ ^]^

**Figure 5:**
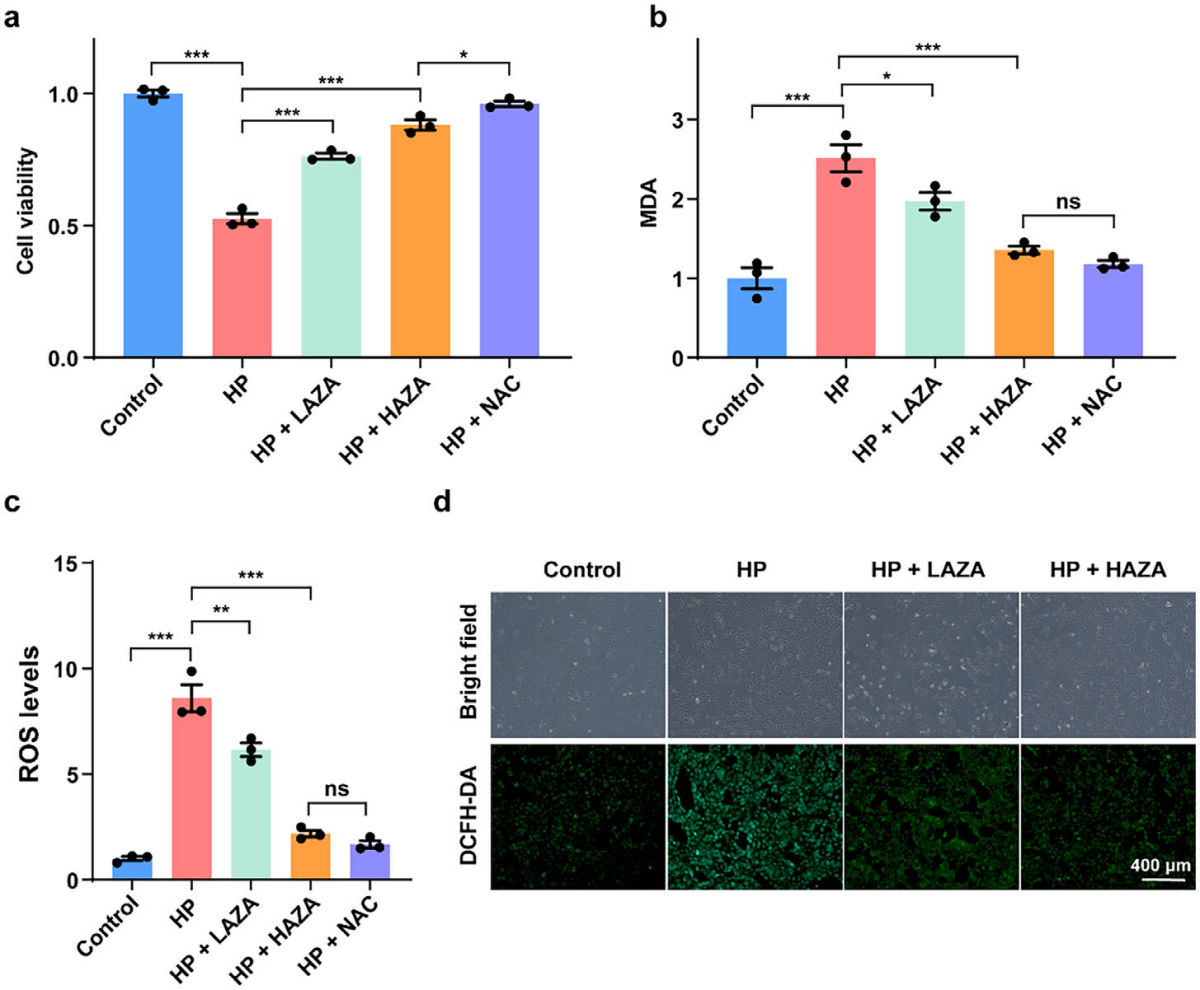
AZA protects ARPE‐19 cells from oxidative stress. a,b) The cell viability (in a) and MDA level (in b) of the ARPE‐19 cells in the control (untreated cells), HP (0.4 mm HP), HP + LAZA (0.4 mm HP + 0.5 mm AZA), HP + HAZA (0.4 mm HP + 5 mm AZA), and HP + NAC (0.4 mm HP + 5 mm NAC) groups. c,d) The ROS level detected by DCFH‐DA staining of the ARPE‐19 cells in those groups. The quantitative fluorescence intensities of ROS levels were in c. The typical fluorescence images were in d (scale bar of 400 µm), and those of the HP + NAC group were in the Supporting Information. Each experiment was performed with three biological replicates, shown by the datapoints in the bar graphs. The data was expressed as the mean ± standard error. *, **, ***, and ns respectively represented p‐values <0.05, p‐value < 0.01, p‐values <0.001, and p‐values > 0.05 in the analysis of variance with Tukey post‐hoc tests.

For activity evaluation of AZA, we chose ARPE‐19 cells as the in vitro cell model, because the malfunction of the retinal pigment epithelium plays a central role in macular degeneration.^[^ ^16^ ^]^ The ARPE‐19 cells were treated with hydrogen peroxide (H₂O₂, HP) to simulate the retinal pigment epithelial cells in HM eyes, which suffer oxidative stress due to the early liquefaction of the vitreous body.^[^ ^17^ ^]^ First, we detected the viability level of ARPE‐19 cells under treatment with different concentrations of HP and found that it showed a dose‐dependent manner (Figure S11, Supporting Information). At a concentration of 0.4 mm, HP reduced the cell viability to about 50% of that of the control group (untreated cells), which hence was selected as the HP treatment concentration for subsequent experiments. Then, we evaluated the inhibitory effect of different concentrations of AZA on HP‐induced cytotoxicity. Interestingly, we found that both the low AZA treatment (LAZA, 0.5 mm) and high AZA treatment (HAZA, 5 mm) could restore the reduction in cell viability of the ARPE‐19 cells caused by HP (p‐values <0.001) with a dose‐dependent manner. Of note, the protective effect of HAZA treatment on cell viability was roughly comparable to the same‐dose N‐acetyl‐L‐cysteine (NAC, 5 mm), a common HP inhibitor, only slightly inferior (p‐value <0.05, less than 0.1‐fold change, **Figure** **5**a).

To evaluate the levels of oxidative damage of ARPE‐19 cells under HP after AZA treatment, we also detected the intracellular level of and malondialdehyde (MDA), a product of lipid peroxidation, and reactive oxygen species (ROS). Consistent with the result regarding cell viability, both the treatment of LAZA and HAZA showed significant protective effects on intracellular oxidative damage (p‐value <0.05 for LAZA, and p‐value <0.001 for HAZA, compared to the HP group, Figure 5b), the latter of which did not show significant differences in comparison with the same‐dose NAC treatment. Further in ROS detection by dichlorodihydrofluorescein‐diacetate (DCFH‐DA) staining, AZA also exhibited the significant capability to scavenge ROS in the HP‐treated ARPE‐19 cells (both the p‐values <0.01) in a dose‐dependent manner (Figure 5c,d; Figure S12, Supporting Information). In sum, the above results indicate that AZA, especially that of a high concentration, can protect cell viability and reduce the cellular oxidative damage of ARPE‐19 cells under oxidative stress, which may play a pivotal role in PM development.

## 3 Discussion

PM, as the detrimental subtype of HM, is one of the leading causes of blindness and has become a major concern of ophthalmologists. Currently, the fundus examination‐based MMD grading and PM diagnosis have matured in clinical practice, which mostly rely on the clear fundus photographs and experience of professional ophthalmologists. However, in most situations, such as there is no fundus photography equipment, or when severe cataracts or corneal diseases prevent clear visualization of the fundus, it is impossible to make a diagnosis of PM by the fundus examination‐based approach. Besides, if the doctor has insufficient experience, it is also difficult to achieve accurate MMD grading and PM diagnosis. Therefore, there is still an unmet clinical need for a highly accessible, objective, and non‐expertise‐dependent PM diagnosis approach with applicability for widespread accurate screening.

In contrast, molecular‐based approaches can offer precise and objective diagnoses without reliance on personal clinical experience, which have become emerging tools in the field of ophthalmic diagnosis.^[^ ^11^, ^18^ ^]^ In terms of MMD grading and PM diagnosis, existing studies have reported some molecular biomarkers in intraocular samples, such as aqueous humor and vitreous humor.^[^ ^19^ ^]^ However, intraocular samples require invasive sampling with limited prospecting for diagnostic applications, highlighting the need for further molecular analysis based on minimally invasive samples. In this respect, our previous works have reported protein array‐based approaches for diagnosing PM using serum and tears.^[^ ^20^ ^]^ Nevertheless, the above protein detection methods have the drawbacks of complex procedures and high costs, making them unsuitable for large‐scale PM screening, which encourages us to further develop the convenient, cost‐effective, and high‐throughput molecular diagnostic approach of PM in the present study.

Herein, using the high‐performance NELDI‐MS, we achieved rapid (<30 s) and in‐depth (200 features) metabolic analysis for 100 nL of plasm samples from 125 HM patients with MMD 1–4. The raw plasm sample only needed a simple tenfold dilution in water before detection, without the reliance on other complex pretreatments. On this basis, we built a diagnostic model of PM through machine learning based on the PMFs. Importantly, the model reached a strong AUC of 0.874 for distinguishing PM from simple HM, corresponding to a sensitivity of 85.7% and a specificity of 70.8%. Further, the model exhibited the capability for diagnosing early‐stage PM, i.e., MMD 2, with a similarly strong AUC of 0.889. Of note, when using only six features as the panel, the model maintained high diagnostic performance (AUC of 0.850), indicating its good potential for clinical translation. In the validation step based on an extra external cohort (123 HM patients with MMD 1–4), the strong AUC of 0.814 demonstrated the reproducibility of the panel's diagnostic performance. Considering the simple sample pretreatment, sub‐minute instrument occupancy time per sample, and sample microarray‐based automatic detection, our molecular diagnostic approach promises a convenient, low‐cost, and high‐throughput tool, with satisfactory precision, for screening PM among large‐scale HM patients, which is difficult to achieve by fundus examination‐based or existing molecular‐based approaches.

Blood metabolic biomarkers represent the signature of systemic metabolic reprogramming,^[^ ^21^ ^]^ which are valuable for molecular mechanism exploration of PM development. Previous works have reported blood metabolic disorders related to myopia or myopic complications. Compared to emmetropia or mild myopia, HM exhibited a close association with metabolic biomarkers, including multiple amino acids, dopamine, melatonin, fatty acids, phospholipids, sphingolipids, and so on.^[^ ^7^, ^22^ ^]^ With regard to PM, a previous study revealed that altered thiamine metabolism and purine metabolism in the blood may be related to macular neovascular PM.^[^ ^23^ ^]^ Nevertheless, in terms of MMD, systemic metabolic reprogramming regarding PM (serious MMD of grades ≥ 2) compared to simple HM (MMD 1) remains to be elucidated. Herein, based on machine learning to parse the PMFs, we identified six biomarkers representing the disordered blood metabolic pattern regarding PM, which included five amino acids and derivatives (Thr, Ala, Gln, Trp, and HBCT), as well as a dicarboxylic fatty acid (AZA). The five amino acids and derivatives indicated dysregulated blood metabolism of amino acids regarding PM, some of which also play key roles in oxidative stress regulation and hormone/neurotransmitter biosynthesis.^[^ ^24^ ^]^ Besides, the changed level of HBCT, an acylcarnitine derivative involving mitochondrial *β*‐oxidation of fatty acids, suggested dysregulated lipid and energy metabolism.^[^ ^13^, ^25^ ^]^ AZA is an antioxidative and anti‐inflammatory fatty acid, which was previously reported as an intraocular biomarker related to myopia.^[^ ^14^, ^15^ ^]^ The correlation analysis revealed close relationships among amino acids, as well as those between AZA and amino acids, suggesting the co‐varied metabolism of amino acids and lipids may be the key metabolic reprogramming in the PM development. In sum, we found that PM was related to systemic reprogramming of amino acid and lipid metabolism, which may mediate dysfunctional oxidative stress, inflammation, hormone/neurotransmitter systems, and energy metabolism, and thus play critical roles in the development of PM. Compared to metabolic biomarkers reported by previous studies focused on myopia,^[^ ^7^, ^22^ ^]^ though the six biomarkers found here were partially different, they indicated similar dysregulated metabolic pathways and abnormal function. The differences in specific biomarkers can be attributed to the following factors: 1) Study design. We for the first time investigated metabolic biomarkers between PM and simple HM, rather than between myopia and non‐myopia. 2) Study methods. We employed an integrative strategy of MS and machine learning to find biomarkers, rather than conventional chemometric approaches, such as principal component analysis, partial least squares‐discriminant analysis.

Although MMD 2–4 is referred to as PM, it is particularly noteworthy that MMD 4 may be completely different from MMD 2–3. Specifically, MMD 1 was defined as a tessellated fundus; MMD 2 as diffuse chorioretinal atrophy, presenting as a yellowish white appearance of the posterior pole; MMD 3 as patchy chorioretinal atrophy, which presents as a grayish white lesion, and does not threaten the macula; MMD 4 as macular atrophy, which presents a grayish white lesion, with macula involvement. The essential pathological feature that distinguishes MMD 4 from MMD 2–3 is severe chorioretinal atrophy located in the macula, the most visually functional area, which was also observed in this study (Figure 2a). In this study, on the basis of the comparison between PM and simple HM, we further revealed the specificity of MMD 4 in this disordered metabolism compared to MMD 2–3, which may be related to its characteristic fundus pathology. Through PMF parsing by machine learning, three biomarkers were determined to represent the specific systemic metabolic reprogramming of MMD 4, including two medium‐chain fatty acids (3‐HDA and AZA) and an amino acid derivative (CT). CT is an essential substrate for producing the acylcarnitine‐fatty acids for mitochondrial import and following energy metabolism, including 3‐HDA and AZA.^[^ ^13^, ^14^ ^]^ While AZA, as an antioxidative monocarboxylic fatty acid, could also participate in CT‐independent pathways for other functions like anti‐oxidation.^[^ ^14^ ^]^ Interestingly, the AZA level was in a decreased trend in the MMD 2–3 group compared to the simple HM group, while this trend was recovered in the MMD 4 group (cluster 3 in Figure 4b). In contrast, 3‐HDA and CT levels were flattened in the MMD 2–3 group compared to the simple HM group but were strongly decreased in the MMD 4 group (cluster 2 in Figure 4b). Consistently, 3‐HDA and CT exhibited a significant correlation in the MMD 2–3 and MMD 4 groups, while AZA did not exhibit significant correlations for those two metabolites. These findings suggested AZA's difference between the MMD 2–3 and MMD 4 groups was more likely related to CT‐independent pathways and other abnormal functions, like the antioxidative system, rather than simple mitochondrial energy metabolism. We provided molecular mechanistic insights underlying the different fundus pathologies between MMD 2–3 and MMD 4 from the perspective of systemic metabolism. Further, considering the differences in systemic metabolic reprogramming and fundus pathology, MMD 4 may not be induced by simple aggravation of MMD 2 and 3, highlighting the specificity of MMD 4.

Of those biomarkers identified here, we performed the bioactivity evaluation for AZA to further investigate its role in MMD development. Using the ARPE‐19 cells with HP treatment, we simulated the oxidative stress status of retinal pigment cells undergoing early liquefaction of the vitreous body in HM eyes. Of interest, we found that AZA could restore HP‐induced decline in cell viability and abnormal oxidative stress in ARPE‐19 cells, suggesting it may be a key bioactive metabolite involving PM development. Of note is that AZA is a reported human corneal biomarker for moderate to high myopia.^[^ ^15^ ^]^ In our study, it was identified as a blood PM biomarker, which indicated the close relation of PM to metabolic reprogramming in both intraocular and blood. And, as an antioxidant active metabolite, the level difference of AZA between MMD 2–3 and MMD 4 suggested the different molecular mechanisms of these two types of PM in oxidative stress. Altogether, our findings suggested the changed blood AZA level may play a crucial role in PM development, and showed the strong promise of systemic metabolism regulation in its interventions.

## 4 Conclusion

In conclusion, by combining NELDI‐MS‐based high‐performance metabolic analysis and machine learning, we constructed a convenient, low‐cost, and high‐throughput blood test‐based PM molecular diagnostic approach with satisfactory precision, suitable for large‐scale screening. We identified six PM‐associated blood metabolic biomarkers, including Thr, Ala, Gln, Trp, AZA, and HBCT, which indicated the systemic reprogramming of amino acid and lipid metabolism. Besides, we also revealed the specificity of MMD 4 in this reprogramming, which the three biomarkers represented (AZA, 3‐HDA, and CT), compared to MMD 2–3, suggesting its pathogenesis specificity. Further, we found AZA may be involved in PM development as a key antioxidant active metabolite. Our work would contribute to PM molecular diagnosis and pathology exploration.

## Conflict of Interest

The authors declare competing financial interests. The authors have filed patents for both the technology and the use of the technology to detect bio‐samples.

## Supporting information



Supporting Information

## Data Availability

The data that support the findings of this study are available from the corresponding author upon reasonable request.
