# Supplementary material for: Plasma Metabolic Profile with Machine Learning Reveals Distinct Diagnostic and Biological Signatures for Pathologic Myopia
Source: Adv Sci (Weinh). 2025 Jul 25;12(39):e05861. doi: 10.1002/advs.202505861 (PMC12533321; doi:10.1002/advs.202505861)
Supplement: Supplementary file 1 — Supporting Information [file ADVS-12-e05861-s001.docx]

Supporting Information

**Plasma Metabolic Profile with Machine Learning Reveals Distinct Diagnostic and Biological Signatures for Pathologic Myopia**

*Ziheng Qi, Jiao Qi, Ye Zhang, Yanhui Wang, Yuchen Feng, Zifan Yang, Yating Wang, Weikang Shu, Dongling Guo, Ching Kang, Keke Zhang, Yi Lu, Jingjing Wan,^*^ and Xiangjia Zhu^*^*

**1. Experimental section**

**1.1. Chemicals and reagents**

The chemicals and reagents in this work included those used in the following respects: (1) the ferric nanoparticle (NP) synthesis; (2) the performance evaluation for the NELDI-MS; and (3) the biological evaluation for azelaic acid. In the ferric NP synthesis, ferric chloride hexahydrate (97%), ethylene glycol (99.5%), trisodium citrate (99%), anhydrous sodium acetate (99%), and absolute ethanol (99.7%) were ordered from Sinopharm Chemical Reagent Beijing Co. Ltd. (Beijing, China). In the performance evaluation for the NELDI-MS, glucose (99.5%), arginine (99%), valine (99%), alanine (99%), AZA (99%), and mannitol (97%) were purchased from Adamas (Shanghai, China). Trifluoroacetic acid (TFA, 99.5%) and acetonitrile (ACN, 99.9%) were obtained from Adamas (Shanghai, China). 2,5-dihydroxybenzoic acid (DHB, 99%), α-cyano-4-hydroxy-cinnamic acid (CHCA, 99%), and bovine serum albumin (98%) were ordered from Sigma-Aldrich (St. Louis, MO, USA). In the biological evaluation for AZA, AZA (99%) was purchased from Adamas (Shanghai, China), hydrogen peroxide (HP, 30%) was purchased from Thermo Fisher Scientific, Inc. (Waltham, USA), and N-acetyl-L-cysteine (NAC, 99.8%) was purchased from MedChemExpress (Monmouth Junction, NJ, USA). Besides, the experiment water used in this work was prepared by the ultrapure water system (18.2 MΩ cm, Milli-Q, Millipore, GmbH).

**1.2 Subjects**

This research was carried out in compliance with the principles outlined in the Declaration of Helsinki and was approved by the Institutional Review Board of the Eye, Ear, Nose, and Throat Hospital of Fudan University, Shanghai, China (No. 2020068). Clinical data and plasma samples used in this research were collected from the Clinical Biological Sample Bank of the Shanghai High Myopia Study. Before recruitment, all subjects in the Shanghai High Myopia Study signed informed consent forms for the use of their clinical data and plasma samples.

This study included high myopia (HM) participants (axial length of bilateral eyes ≥ 26 mm) with different myopic macular degeneration (MMD) grades in the Shanghai High Myopia Study. The 125 patients in the main cohort were recruited between December 2022 and June 2024 (the demographic characteristics were in Table S1), and the 123 patients in the extra external cohort were recruited between September 2024 and May 2025 (the demographic characteristics were in Table S4). The exclusion criteria were as follows: (1) other retinopathies, such as retinal vasculitis, retinal detachment, retinal vein occlusion, macular holes, etc.; (2) other ocular diseases, such as glaucoma and uveitis; (3) previous ocular trauma or surgery; (4) systemic metabolic diseases, such as hyperthyroidism, hypothyroidism, and diabetes mellitus; (5) a history of tumors; (6) a treatment history of medication in recent six weeks. Participants without fundus photographs or with unclear images that did not allow for the grading of MMD were also excluded.

The fundus photographs of participants in this study were all acquired using ultra-widefield photography (Optos 200Tx, Optos, England). The Meta-Analysis for Pathologic Myopia (META-PM) study group developed a classification system for MMD, which is widely recognized and extensively used in various studies. In this study, we also based our classifications on the META-PM system and identified the MMD grades of HM eyes, assigning them a grade from 1 to 4 according to the fundus photographs. Two experienced doctors, J. Qi and K. Zhang, independently graded the fundus photographs of HM eyes. In case of any discrepancy between the two doctors, a senior eye specialist, XZ, provided the final adjudication. MMD 1 was classified as simple HM, and MMD 2-4 was classified as PM. The MMD grade for HM subjects was determined based on the grade of the worse eye.

**1.3 Collection of plasma samples**

Participants in the Shanghai High Myopia Study had 1 ml of venous blood routinely collected per subject using anticoagulation vacuum blood tubes. Blood samples need to be processed within 4 hours after collection. The top plasma layer was aspirated into test tubes after centrifugation at 3000 rpm for 10 minutes at 4 °C. All plasma samples were stored at -80 °C until used.

**1.4 Ferric NP synthesis**

A low-cost solvothermal method was employed to prepare the ferric NPs, following the previous work. First, the ferric chloride hexahydrate, anhydrous sodium acetate, and trisodium citrate were added to the ethylene glycol solution. Next, the solution was sonicated at 25°C for 45 minutes, and then was transferred to the autoclave for hydrothermal reaction at 200 °C for 10 h. Finally, the product was washed with ethanol and dried for further use. In terms of characterization, the X-ray diffraction analysis was performed using a D8 Advanced X-Ray Diffractometer (Bruker, Germany), the transmission electron microscopy and elemental mapping images were obtained using a JEOL JEM-2100F instrument (JEOL Ltd., Japan), and the scanning electron microscopy images were obtained using an S4800 field emission scanning electron microscope (Hitachi, Japan).

**1.5 NELDI-MS detection**

The nanoparticle-enhanced laser desorption/ionization MS (NELDI-MS) detection was performed on Bruker Autoflex (time-of-flight MS, used in metabolic fingerprinting) and Solarix 7.0T (Fourier transform ion cyclotron resonance -MS, used in metabolic annotation) equipped with a Nd:YAG laser of 355 nm. The samples were either 10-fold water dilution of plasma or standard metabolites at 1mg mL^-1^. The matrices were 1 mg mL^-1^ of the ferric NPs (dispersed in water), 10 mg mL^-1^ of DHB in TA30 (30% ACN/70% water, 0.1% TFA), or saturation concentration of CHCA in TA30. Before MS detection, 1 μL of the sample was loaded onto the NELDI-MS chip to form a dried sample spot under ambient conditions. Then, 1 μL of the matrix was loaded to cover the sample spot. The MS data was automatically acquired from the microarrayed sample spots on the chip with laser shots of 2000. For each sample, the detection was independently repeated five times to minimize random error (these replicates could be finished within 30 seconds). To correct the mass axis and ensure data reliability, a solution of mixed standards was tested at a regular interval.

**1.6 NELDI-MS data processing and machine learning**

The raw MS data was processed using a home-built Python script based on the OpenMS package, including steps of peak detection, alignment, filtration, and normalization. The fingerprint of each sample represented the average spectrum of the five repeated detections.

The machine learning was performed using the sk-learn scientific computing framework. The data in the discovery cohort was employed in the hyperparameter tuning (by the k-fold cross-validation, k = 3), model building, and the determination of the biomarker panels. The data in the validation cohort was not involved in the above process and was only used for validating model performance and avoiding overfitting risk. The feature panel was further validated by the data in the external cohort to evaluate the reproducibility.

**1.7 LC-MS/MS analysis**

For sample preparation, 400 μL of methanol/acetonitrile (50/50, v/v) solution was added to 100 μL of the representative plasma sample (equally mixing six samples per group) and was vortexed for 1 min. Next, the mixture was placed at -20 °C for 2 hours, and then centrifuged for 20 min at 13000 rpm. The supernatant was collected, dried by centrifugation at 4 °C, and redissolved in 150 μL of methanol/water (30/70, v/v) for LC-MS/MS analysis.

A ZenoTOF 7600 (SCIEX, USA) UPLC-QTOF system was employed to carry out the LC-MS/MS analysis. The chromatographic separation was performed using an LC-20A (Shimadzu, Japan) system, with an HSS T3 column (particle size, 1.8 μm; 100 mm (length) × 2.1 mm (i.d.)) at the oven temperature of 40 °C. The mobile phase A was 0.1% formic acid in water, and the mobile phase B was pure acetonitrile. The gradient system was set at 0-0.4 min: 2% B, 0.4-6 min: 2%-25% B, 6-16 min: 25%-100% B, 16-19 min: 100% B, 19-19.1 min: 100%-2% B, 19.1-23 min: 2% B, with an injection of 5 μL and a flow of 0.3 mL min^-1^. For MS parameters, both the positive and negative electrospray ionization mode was performed. The MS range was set at 50-1000 Da, and the MS/MS range was set at 50-500 Da. An IDA acquisition mode was employed with a maximum of 25 of MS/MS experiments per cycle. The MS/MS experiments were performed using CID fragmentation with a collision energy of 35 V for positive mode and -35 V for negative mode, and the energy spread was set at 15 V.

**1.8 Cell culture and treatment**

A retinal epithelial cell line, ARPE-19 cell (serial of GNHu45 from the National collection of authenticated cell cultures of China, male human origin), was employed in this study. It is a differentiated retinal pigment epithelium (RPE) cell line, which expresses RPE-specific molecular markers such as cellular retinaldehyde-binding protein (CRALBP) and RPE65. The ARPE-19 cells were routinely cultured in F-12 medium (G4610, Servicebio) supplemented with 10% fetal bovine serum (G8003, Servicebio) and 1% streptomycin mixture (G4003, Servicebio), and maintained at 37 ^◦^C under 5% CO_2_. To evaluate the effect of AZA on cell viability under oxidative stress, cells were pretreated with the desired concentrations of AZA or a common HP inhibitor, NAC, for 1 hour, followed by treatment with 0.4mM HP for 24 hours. When assessing the impact of AZA on the cellular oxidative damage levels under oxidative stress, cells were also pretreated with the same concentrations of AZA or 5mM NAC for 1 hour, followed by 0.4mM HP treatment for just 1 hour.

**1.9 Cell viability assay**

Cell viability was measured using a Cell Counting Kit-8 (CCK-8, BS350E, Biosharp) according to the manufacturer’s protocol. After treatment under different conditions in a 96-well plate, 10 μL CCK8 was subsequently added to each plate and incubated for 40 min at 37 ^◦^C under 5% CO_2_. Cell viability was read as the absorbance at wave length of 450 nm, using a microplate reader (Molecular Devices, Tecan Spark). Cell viability was expressed as a ratio compared to untreated control cells.

**1.10 Assessment of intracellular oxidative damage levels**

We used Reactive Oxygen Species Assay Kit (S0033, Beyotime) to assess the levels of intracellular reactive oxygen species (ROS). Briefly, after exposure to different concentrations of AZA or 5mM NAC for 1 hour followed by 0.4mM HP 1h, ARPE-19 cells were further treated with 1:1000-diluted 2,7-dichlorodihydrofluorescein diacetate (DCFH-DA) at 37°C under light-protected conditions for 30 minutes. ROS levels were determined by detecting the fluorescence intensity of the oxidized DCF using flow cytometry (BD FACSCelesta, USA) on collected single-cell suspensions. For adherent cells subjected to the same treatment, fluorescence images of DCFH-DA-stained cells cultured in culture dishes were observed under a fluorescence microscope (Carl Zeiss, Oberkochen, Germany). In parallel, after treatment under different conditions, the ARPE-19 cells were collected and washed. Then, they were also lysed, and the supernatants were sucked out. The malondialdehyde (MDA) levels were detected, which is a product of lipid peroxidation and can reflect the level of oxidative damage, according to the manufacturer's protocol (A003-4-1, Nanjing Jiancheng Bioengineering Institute). fluorescence intensity of DCF and level of MDA content were expressed as a ratio compared to untreated control cells.

**1.11 Statistical analysis**

The information regarding data presentation, sample size for each statistical analysis, statistical methods, testing level, and significance annotations was included in the figure legends. The two-tailed t-test and one-way analysis of variance (including Tukey post-hoc tests) were used to examine the significance of differences in two-group mean comparisons and multiple-group mean comparisons, respectively (significance level was set at 0.05). The *χ*2 test was used to determine the significance of sex differences (sex assigned at birth) in the cohort (significance level was set at 0.05). The p-values for those were calculated by GraphPad Prism 10.0, SPSS v22.0. The Pearson's correlation coefficients among the independent repeated detections for the representative plasma sample were obtained by Origin 2025. The Kendall's correlation analysis for the biomarkers was performed by Origin 2025 (significance level was set at 0.1). The median normalization was applied to the NELDI-MS data, which was next scaled to 0 to 1 before machine learning. The area under the curve (AUC), sensitivity, specificity, corresponding 95% CI, and p-value of the machine learning models were calculated using the SK-Learn scientific computing framework and GraphPad Prism 10.0. Delong tests were employed to examine the significance of AUC. The power analysis, the dimensionality reduction chemometric analysis, and the unsupervised clustering for discriminating the PMFs of different grade MMD were performed by MetaboAnalyst (https://www.metaboanalyst.ca/). The clustering analysis for the 200 features within the PMFs to identify their typical changing trends was performed using a home-built R script based on the Mfuzz package.

**2. Supplemental figure**


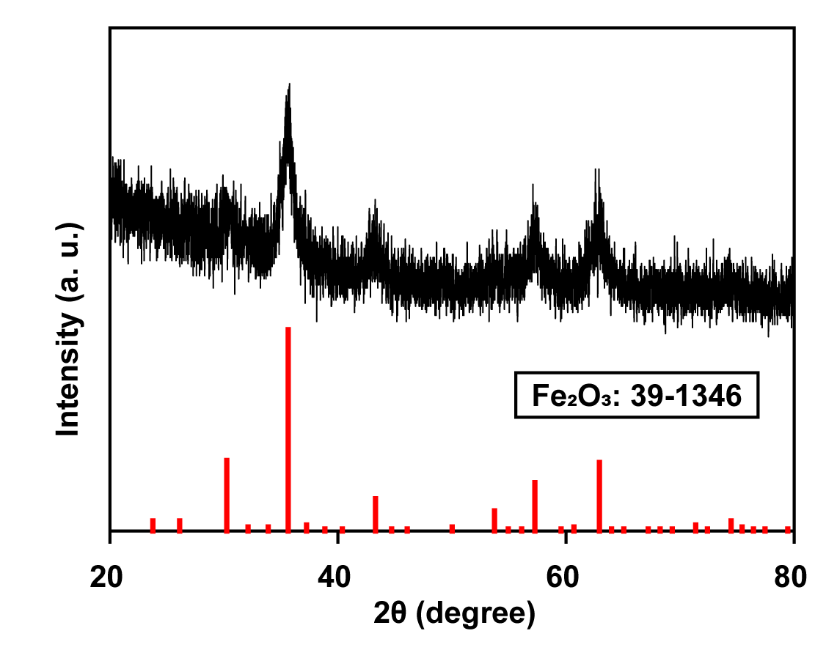


**Figure S1.** X-ray diffraction of the ferric NPs (upper graph) and JCPDS:39-1346 of Fe_2_O_3_ (lower graph).


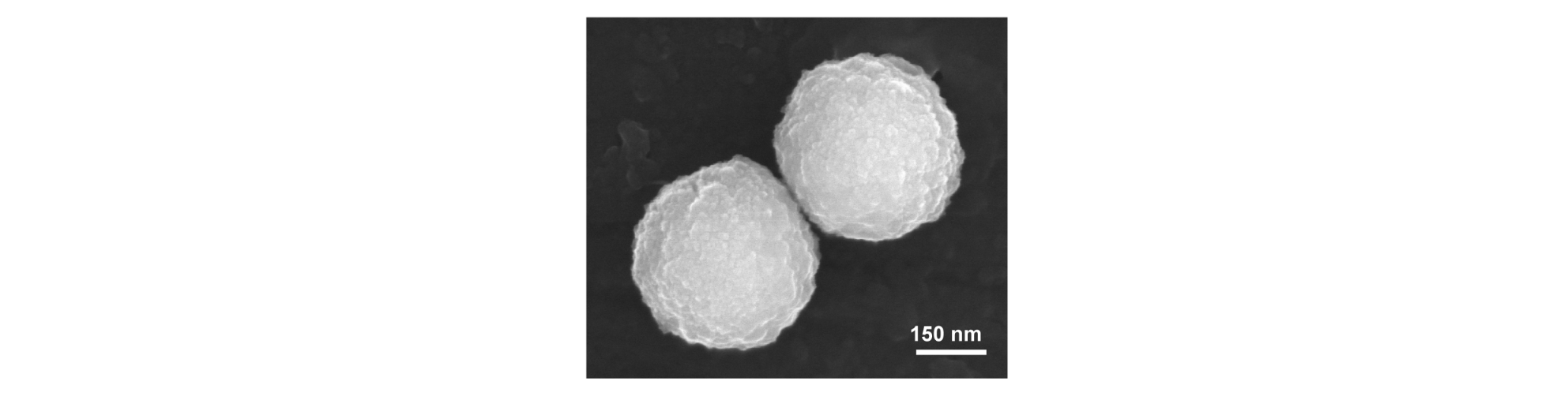


**Figure S2.** The scanning electron microscopy image of the NPs (scale bar of 150 nm).


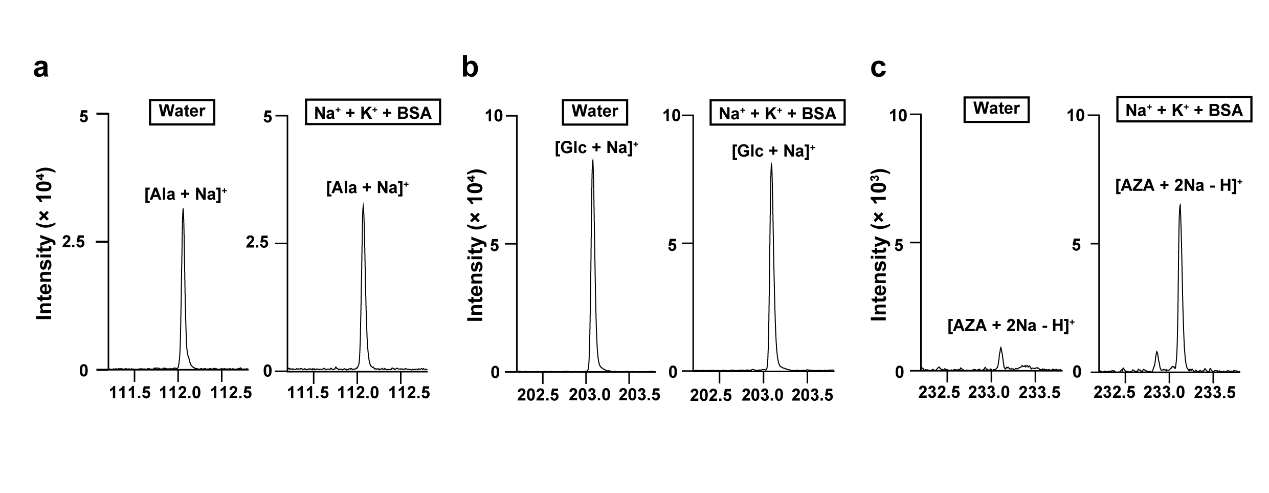


**Figure S3.** NELDI-MS detection for 1mg ml^-1^ alanine (in a), glucose (in b), and azelaic acid (in c) in a water solution and a mixed solution of high salt and protein (15 mM of Na^+^, 0.5 mM K^+^, and 10 mg mL^-1^ of protein of bovine serum albumin). The signals of the test metabolites were annotated in the figures. Ala, alanine, Glc, glucose, AZA, azelaic acid, BSA, bovine serum albumin.


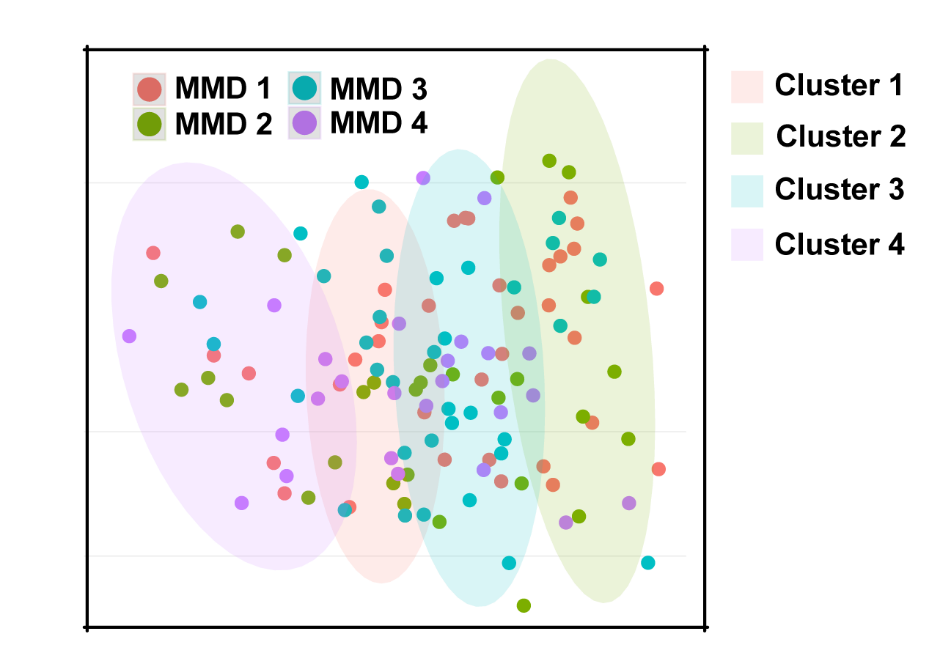


**Figure S4.** The unsupervised clustering for the PMFs. The K-means clustering process resulted in four clusters, which failed to separate the PMFs of different grade MMD.


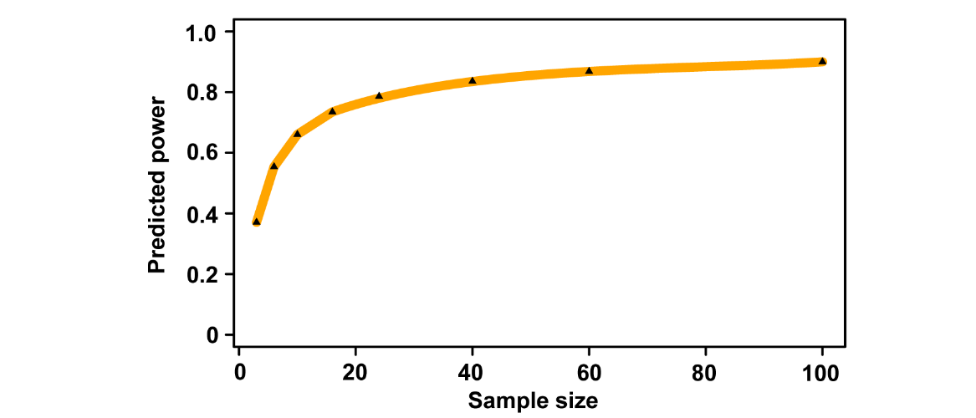


**Figure S5.** Power analysis of the pilot data (simple HM/PM, 5/5). The FDR was set at 0.15.


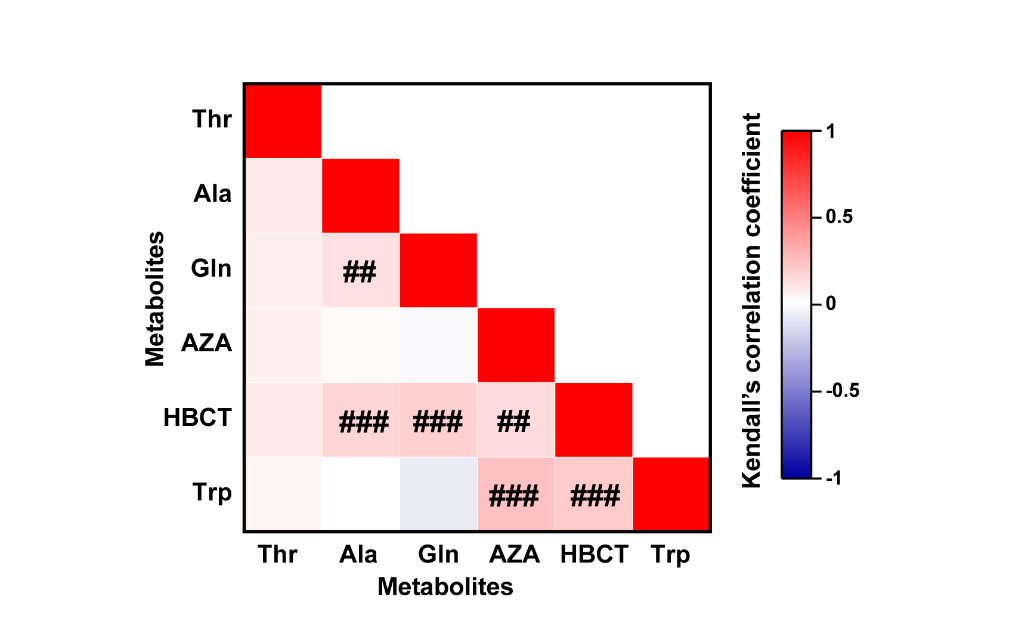


**Figure S6.** The Kendall's correlation analysis for the six biomarkers for discriminating PM from simple HM. The significance level for correlation analysis was set at 0.1. The ## and ### represented p-values < 0.05 and 0.01, respectively.


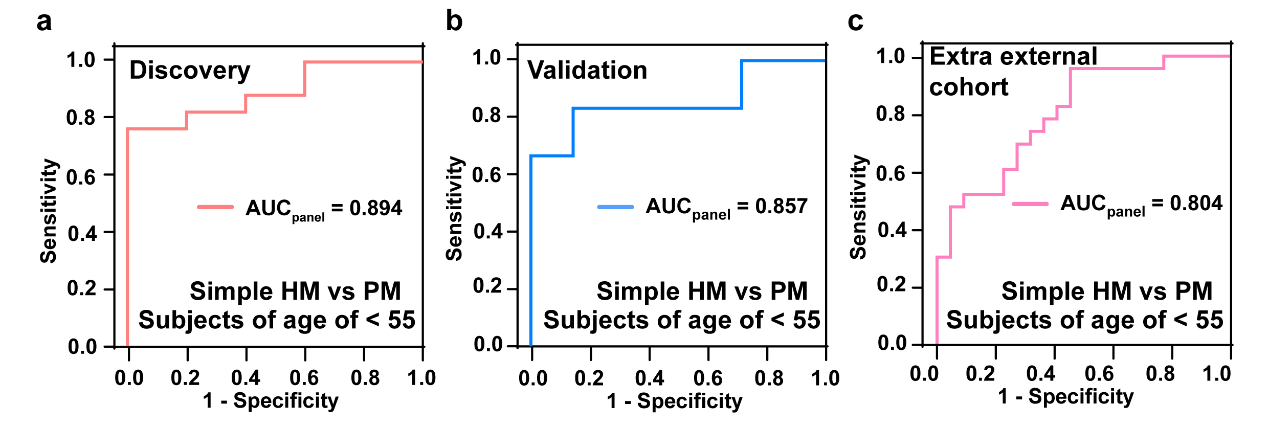


**Figure S7.** The ROC curves by the biomarker panel-based LR model for discriminating PM from simple HM in the stratifications of subjects with an age less than 55 years. The stratified discovery AUC was in a (simple HM/PM, 5/17), the stratified validation AUC was in b (simple HM/PM, 7/6), and the AUC in the stratified extra external cohort was in c (simple HM/PM, 22/23).


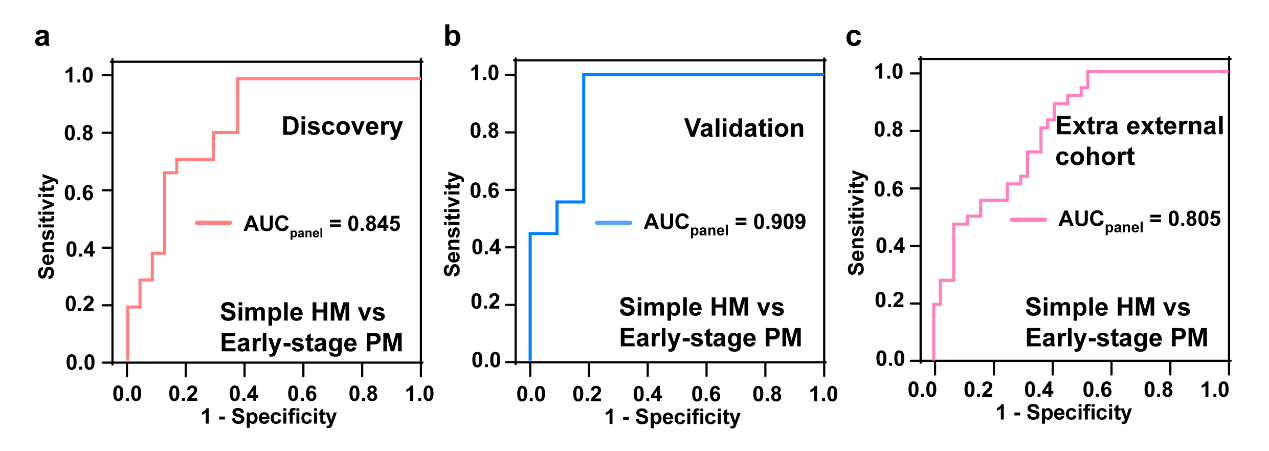


**Figure S8.** The ROC curves by the biomarker panel-based LR model for discriminating early-stage PM from simple HM. The discovery AUC was in a (simple HM/early-stage PM, 24/21), the validation AUC was in b (simple HM/early-stage PM, 11/9), and the AUC in the extra external cohort was in c (simple HM/early-stage PM, 44/36).


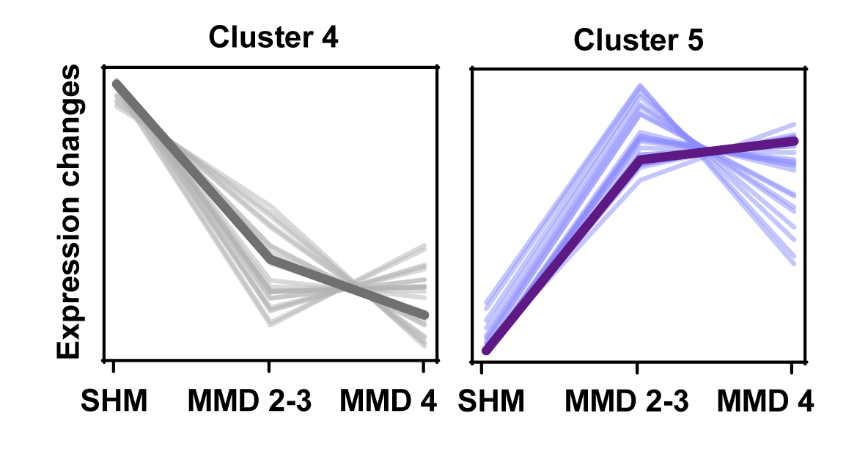


**Figure S9.** The clusters each represented a typical type of change trend of the features. Two out of the five clusters were shown here, while the other three clusters were shown in Figure 4b.


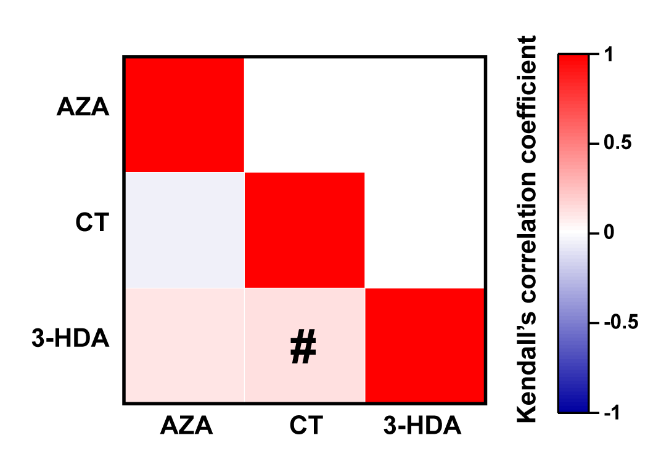


**Figure S10.** The Kendall's correlation analysis for the three biomarkers for discriminating MMD 4 from MMD 2-3. The significance level for correlation analysis was set at 0.1. The # represented p-value < 0.1.


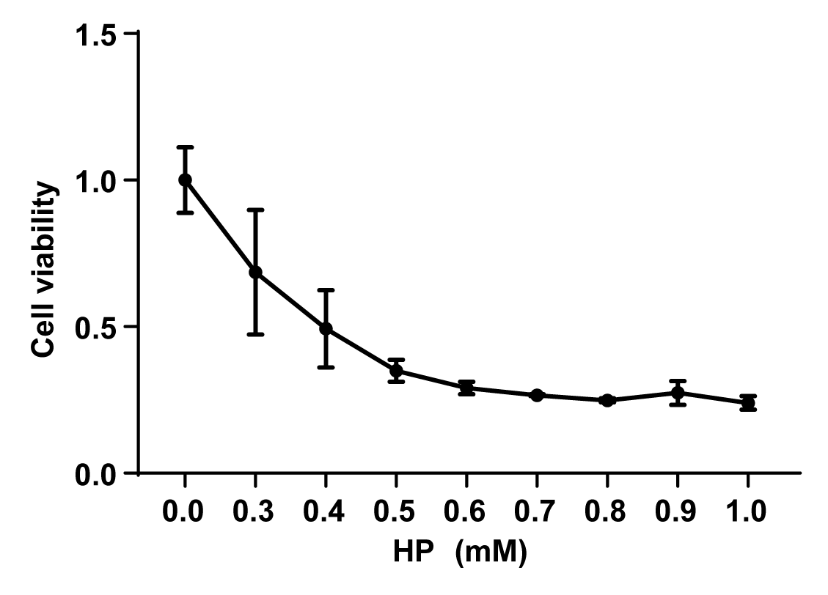


**Figure S11.** The viability level of ARPE-19 cells under treatment with different concentrations of HP.


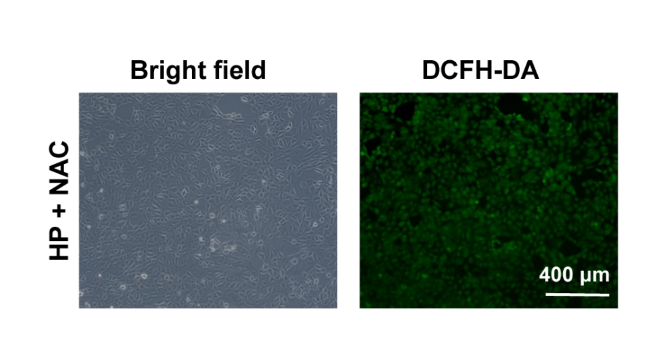


**Figure S12.** The typical fluorescence image of the ARPE-19 cells in the HP + NAC group in the ROS level detection by DCFH-DA staining (scale bar of 400 μm).

**3. Supplemental table**

**Table S1.** **Demographic characteristics of the participants in the main cohort (n = 125).**

|  | Myopic macular degeneration grades | | | | P-value^a)^ |
| --- | --- | --- | --- | --- | --- |
|  | MMD 1 | MMD 2 | MMD 3 | MMD 4 |  |
| Num | 35 | 30 | 35 | 25 | / |
| Age, years, ± SD | 59.5 ± 10.7 | 62.0 ± 10.4 | 61.0 ± 9.0 | 61.2 ± 11.8 | 0.795 |
| Sex, male/female^b)^ | 15/20 | 12/18 | 10/25 | 7/18 | 0.485 |
| Axial length,  mm, ± SD | 28.74 ± 1.59 | 30.19 ± 1.53 | 31.08 ± 2.61 | 30.93 ± 2.31 | 0.000 |

^a)^ Comparison among groups was analyzed using the one-way analysis of variance (continuous data) or *χ*2 test (categorical data).

^b)^ Sex referred to the sex assigned at birth.

**Table S2. The six features in the panel for discriminating the PM group and the simple HM group.**

| No. | m/z in  NELDI-MS | Metabolite | Ion adduction | Fold change^a)^ | P-value^b)^ |
| --- | --- | --- | --- | --- | --- |
| 1 | 102.1 | Threonine | [M + H - H_2_O]^+^ | 1.26 | 0.016 |
| 2 | 112.0 | Alanine | [M + Na]^+^ | 0.83 | 0.047 |
| 3 | 169.0 | Glutamine | [M + Na]^+^ | 0.89 | 0.024 |
| 4 | 233.2 | Azelaic acid | [M + 2Na - H]^+^ | 0.79 | 0.011 |
| 5 | 268.0 | Hydroxybutyrylcarnitine | [M + K - H_2_O]^+^ | 0.89 | 0.022 |
| 6 | 281.0 | Tryptophan | [M + 2K - H]^+^ | 0.88 | 0.031 |

^a)^ Calculated by the ratio of the PM group to the simple HM group.

^b)^ Calculated by two-tailed t-test.

**Table S3 The LC-MS/MS validation for the biomarker panel for discriminating the PM group and the simple HM group.**

| No. | Metabolite | Chemical formula | Ion adduction in LC-MS/MS | Retention  time (sec) | m/z in LC-MS/MS | Main MS/MS fragments |
| --- | --- | --- | --- | --- | --- | --- |
| 1 | Threonine | C_4_H_9_NO_3_ | [M + H]^+^ | 58 | 120.065 | 56.0, 58.1, 74.1, 84.0, 102.1 |
| 2 | Alanine | C_3_H_7_NO_2_ | [M + K- H_2_O]^+^ | 47 | 110.007 | 66.0, 128.0 |
| 3 | Glutamine | C_5_H_10_N_2_O_3_ | [M + H]^+^ | 56 | 147.076 | 84.1, 101.1, 130.1 |
| 4 | Azelaic acid | C_9_H_16_O_4_ | [M - H]^-^ | 540 | 187.097 | 95.1, 97.1, 123.1, 125.1, 143.1, 169.1 |
| 5 | Hydroxybutyryl-carnitine | C_11_H_21_NO_5_ | [M + H]^+^ | 205 | 248.150 | 85.0, 189.1, 248.1 |
| 6 | Tryptophan | C_11_H_12_N_2_O_2_ | [M + H]^+^ | 329 | 205.097 | 118.1, 144.1, 146.1, 159.1, 188.1 |

**Table S4. Demographic characteristics of the participants in the extra external cohort (n = 123).**

|  | Myopic macular degeneration grades | | | | P-value^a)^ |
| --- | --- | --- | --- | --- | --- |
|  | MMD 1 | MMD 2 | MMD 3 | MMD 4 |  |
| Num | 44 | 36 | 25 | 18 | / |
| Age, years, ± SD | 55.3 ± 11.3 | 56.5 ± 10.2 | 61.6 ± 7.7 | 63.3 ± 10.5 | 0.010 |
| Sex, male/female^b)^ | 20/24 | 12/24 | 11/14 | 5/13 | 0.481 |
| Axial length,  mm, ± SD | 27.36 ± 0.92 | 29.61 ± 1.80 | 31.66 ± 1.70 | 31.41 ± 2.02 | 0.000 |

^a)^ Comparison among groups was analyzed using the one-way analysis of variance (continuous data) or *χ*2 test (categorical data).

^b)^ Sex referred to the sex assigned at birth.

**Table S5. The three features in the panel for discriminating the MMD 4 group and the MMD 2-3 group.**

| No. | m/z in  NELDI-MS | Metabolite | Ion adduction | Fold change^a)^ | P-value^b)^ |
| --- | --- | --- | --- | --- | --- |
| 1 | 233.2 | Azelaic acid | [M + 2Na - H]^+^ | 1.27 | 0.042 |
| 2 | 238.1 | Carnitine | [M+2K-H]^+^ | 0.88 | 0.008 |
| 3 | 239.1 | 3-Hydroxydodecanoic acid | [M + Na]^+^ | 0.94 | 0.047 |

^a)^ Calculated by the ratio of the MMD 4 group to the MMD 2-3 group.

^b)^ Calculated by two-tailed t-test.

**Table S6 The LC-MS/MS validation for the biomarker panel for discriminating the MMD 4 group and the MMD 2-3 group.**

| No. | Metabolite | Chemical formula | Ion adduction in LC-MS/MS | Retention  time (sec) | m/z in LC-MS/MS | Main MS/MS fragments |
| --- | --- | --- | --- | --- | --- | --- |
| 1 | Azelaic acid | C_9_H_16_O_4_ | [M - H]^-^ | 540 | 187.097 | 95.1, 97.1, 123.1, 125.1, 143.1, 169.1 |
| 2 | Carnitine | C_7_H_15_NO_3_ | [M + H]^+^ | 81 | 162.112 | 58.1, 60.1, 85.0, 98.1, 102.1, 103.0, 116.1 |
| 3 | 3-Hydroxy-dodecanoic acid | C_12_H_24_O_3_ | [M - H]^-^ | 740 | 215.165 | 59.0, 215.2 |
